# Music composers and bipolar disorders: Where do we stand?

**DOI:** 10.1192/j.eurpsy.2021.537

**Published:** 2021-08-13

**Authors:** V. Giannouli

**Affiliations:** Institute Of Neurobiology, Bulgarian Academy of Sciences, Sofia, Bulgaria

**Keywords:** Creativity, Bipolar Disorders, Music

## Abstract

**Introduction:**

The intersection between bipolar disorders and creativity has been investigated in western literature. Although psychopathology has been proposed for famous artists in painting, writing, music and other forms of art, there is no systematic study examining bipolar disorders and music production in composers.

**Objectives:**

The aim of this review is to investigate this relationship by providing an overview of published studies.

**Methods:**

The search included papers published in English as abstracts as well as in full length until October 2020. The literature search was conducted using the MEDLINE, EMBASE, PUBMED and GOOGLE SCHOLAR databases. For all the searches, the terms/key words that were used were “bipolar disorder”, “music”, “creativity”, and “composers”.

**Results:**

Search results for composers from different music genres and musical periods indicated that the proposed origin of the overall bipolar pattern is attributed to several stressful environmental factors which are taking the form of interpersonal problems regarding the expressed emotion, life events, and paucity of stress-management skills. In addition to that, bipolar psychopathological patterns seem to influence the quantity of music composing activity indirectly due to changes in everyday functional abilities.

**Conclusions:**

Published reports, although based on biographical research, do provide evidence in support of a strong bipolarity-music creativity/production link for famous composers. Further well-designed studies in living music professionals engaged in music composition are needed.

